# The topology of higher-order complexes associated with brain hubs in human connectomes

**DOI:** 10.1038/s41598-020-74392-3

**Published:** 2020-10-14

**Authors:** Miroslav Andjelković, Bosiljka Tadić, Roderick Melnik

**Affiliations:** 1 Department of Theoretical Physics, Jožef Stefan Institute, 1000 Ljubljana, Slovenia; 2 Complexity Science Hub, Josefstaedter Strasse 39, Vienna, Austria; 3 Department of Thermal Engineering and Energy, Vinča Institute of Nuclear Sciences - National Institute of the Republic of Serbia, University of Belgrade, 1100 Belgrade, Serbia; 4 MS2Discovery Interdisciplinary Research Institute, M2NeT Laboratory and Department of Mathematics, Wilfrid Laurier University, 75 University Ave. W, Waterloo, ON N2L 3C5 Canada; 5 BCAM - Basque Center for Applied Mathematics, Alameda de Mazarredo 14, 48009 Bilbao, Spain

**Keywords:** Neuroscience, Mathematics and computing, Physics

## Abstract

Higher-order connectivity in complex systems described by simplexes of different orders provides a geometry for simplex-based dynamical variables and interactions. Simplicial complexes that constitute a functional geometry of the human connectome can be crucial for the brain complex dynamics. In this context, the best-connected brain areas, designated as hub nodes, play a central role in supporting integrated brain function. Here, we study the structure of simplicial complexes attached to eight global hubs in the female and male connectomes and identify the core networks among the affected brain regions. These eight hubs (Putamen, Caudate, Hippocampus and Thalamus-Proper in the left and right cerebral hemisphere) are the highest-ranking according to their topological dimension, defined as the number of simplexes of all orders in which the node participates. Furthermore, we analyse the weight-dependent heterogeneity of simplexes. We demonstrate changes in the structure of identified core networks and topological entropy when the threshold weight is gradually increased. These results highlight the role of higher-order interactions in human brain networks and provide additional evidence for (dis)similarity between the female and male connectomes.

## Introduction

Recent advances in the science of complex systems aim for a better understanding of the higher-order connectivity as a possible basis for their emerging properties and complex functions. Beyond the framework of pairwise interactions, these connections described by simplexes of different sizes (triangles, tetrahedra and larger cliques) provide the geometry for higher-order interactions and simplex-related dynamical variables. One line of research consists of modelling and analysis of the structure of simplicial complexes in many complex systems, ranging from the human connectome^1^ to quantum physics^2^ and materials science^3,4^. Meanwhile, considerable efforts aim at understanding the impact of geometry on the dynamics. In this context, the research has been done on modelling of the simplex-based synchronisation processes^5,6^, on studying the related spectral properties of the underlying networks^7,8^, as well as on the interpretation of the dynamics of the brain^9–11^ and other complex dynamical systems^12^.

Recently, mapping the brain imaging data^13,14^ to networks involved different types of signals across spatial and temporal scales; consequently, a variety of structural and functional networks have been obtained^15–18^. This network mapping enabled getting a new insight into the functional organisation of the brain^19,20^, in particular, based on the standard and deep graph theoretic methods^21–23^ and the algebraic topology of graphs^1,24^. The type of network that we consider in this work is the whole-brain network *human connectome*; it is mapped from the diffusion tensor imaging data available from the human connectome project^25^, see Methods. The network nodes are identified as the grey-matter anatomical brain regions, while the edges consist of the white-matter fibres between them. Beyond the pairwise connections, current research focuses on the higher organised patterns that may have emerged through the evolutionary optimisation of the relationship between brain structure and function^26^. In this context, researchers described the “rich-club” organisation of important brain regions^27^ and mesoscopic community structure corresponding to typical anatomical brain modules^28^. Furthermore, above the level of the graph, the hierarchical architecture of these modules in the human connectome exhibits a rich structure of simplicial complexes and short cycles between them, as it was shown in^1^. It has been recognised^28,29^ that every module has an autonomous function, which contributes to performing complex tasks of the brain. Meanwhile, the integration of this distributed activity and transferring of information between different modules is performed by very central nodes (hubs) as many studies suggest, see a recent review^11^ and references therein. Formally, hubs are identified as a group of four or five nodes in each brain hemisphere that appear as top-ranking according to the number of connections or another graph-centrality measure. Almost all formal criteria give the same set of nodes, which are anatomically located deep inside the brain, through which many neuronal pathways go. Recently, there has been an increased interest in the research of the hubs of the human connectome. The aim is to decipher their topological configuration and how they fulfil their complex dynamic functions. For example, it has been recognised that the brain hubs are mutually connected such that they make a so-called “rich club” structure^27^. Moreover, their topological configuration develops over time from the prenatal to childhood and adult brain^30,31^. The hubs also can play a crucial role in the appearance of diseases when their typical configuration becomes destroyed^32^. Within another branch of the research, based on the brain imaging data and network mapping, there is growing evidence of the sex-related differences in human connectomes^33–39^. Considering the consensus female and male connectomes, better connectivity in the female connectome has been documented both by the deep-graph theory measures^23^ and by the analysis of simplicial complexes^1^.

In this work, we examine the organisation of simplicial complexes in the *core networks* consisting of hubs and all simplexes attached to them in the human connectomes. We assume that the higher-order connectivity between the brain hubs and other regions involved in these simplicial complexes through weighted edges may provide a clue of how the hubs perform their function. Another open question in this context concerns the potential differences between the female and male connectomes occurring at the level of core networks as compared to the whole brain. Based on our work^1^, we use the consensus connectomes that we have generated at the Budapest connectome server^40,41^. These are connectomes that are common for one hundred female subjects (F-connectome) and similarly for one hundred male subjects (M-connectome), see Methods. Accordingly, we determine the hubs as eight top-ranking nodes in the whole connectome, performing the ranking according to the number of simplexes of all orders in which the node participates. These are the Putamen, Caudate, Hippocampus and Thalamus-Proper in the left and similarly in the right brain hemisphere; they also appear as hubs according to several other graph-theory measures. We then construct core networks around eight leading hubs in both female and male connectomes. We determine the simplicial complexes and the related topological entropy in these core structures. To highlight the weight-related heterogeneity of connections, the structure of these core networks is gradually altered by increasing the threshold weight above which the connections are considered as significant. We show that the connectivity up to the 6th order remains in both connectomes even at a high threshold. Meanwhile, the identity of edges and their weights appear to be different in the F- and M-connectomes.

## Methods

### Data description

We use the data for two *consensus connectomes* that we have generated in^1^ at the Budapest connectome server 3.0^40,41^ based on the diffusion MRI data from Human Connectome Project (HCP) for 500 individuals^25^. The server uses brain mapping tools for for parcellation, tractography, and graph construction^13,42,43^ to map the experimental data of each individual. Then the consensus connectome is determined as a set of edges that are common to a selected group of individuals. As described in^41^, we can select the size of the group and the biological sex of individuals as well as several other parameters, e.g., the number of fibres launched in the tractography phase, resulting in a different outcome. Specifically, with the appropriate settings at the server, we determine the weighted whole-brain networks that are common for 100 female subjects, *F-connectome*, and similarly, *M-connectome*, which is common for 100 male subjects. Each connectome consists of $N=1015$ nodes annotated as the anatomical brain regions, and weighted edges, whose weight is given by the number of fibres between the considered pair of brain regions normalised by the average fibre length. Here, we consider the largest number $10^6$ fibres tracked and set the minimum weight to four. The corresponding core networks *Fc-network* and *Mc-network* are defined as subgraphs of the F- and M-connectomes, respectively, containing the leading hubs and their first neighbour nodes as well as all edges between these nodes. Meanwhile, the hubs are determined according to the topological dimension criteria, as described below and in Results.

### Topology analysis and definition of quantities

We apply the Bron–Kerbosch algorithm^44^ to analyse the structure of simplicial complexes, i.e., clique complexes, in the core Fc- and Mc- connectomes. In this context, a *simplex* of order *q* is a full graph (clique) of $q+1$ vertices $\sigma _q=\left\langle i_0,i_1,i_2,...,i_{q}\right\rangle$. Then a simplex $\sigma _r$ of the order $r<q$ which consists of *r* vertices of the simplex $\sigma _q$ is a *face* of the simplex $\sigma _q$. Thus, the simplex $\sigma _q$ contains faces of all orders from $r=0$ (nodes), $r=1$ (edges), $r=2$ (triangles), $r=3$ (tetrahedrons), and so on, up to the order $r=q-1$. A set of simplexes connected via shared faces of different orders makes a *simplicial complex*. The order of a simplicial complex is given by the order of the largest clique in this complex, and $q_{max}$ is the largest order of all simplicial complexes. Having the adjacency matrix of the graph, with the algorithm, we build the incidence matrix ${\Lambda }$, which contain IDs of all simplexes and IDs of nodes that make each simplex. With this information at hand, we compute three structure vectors^45,46^ to characterise the architecture of simplicial complexes:

- The *first structure vector (FSV):* ${\mathbf {Q}}=\{Q_0,Q_1,\ldots Q_{q_{max}-1}, Q_{q_{max}}\}$, where $Q_q$ is the number of *q*-connected components;
- The *second structure vector (SSV):* $\mathbf {N_s}=\{n_0,n_1, \ldots n_{q_{max}-1},n_{q_{max}}\}$, where $n_q$ is the number of simplexes from the level *q* upwards;
- The *third structure vector (TSV):* the component ${\hat{Q}}_q \equiv 1-{Q_q}/{n_q}$ quantifies the degree of connectedness among simplexes *at* the topology level *q*.

Furthermore, we determine the topological dimension of nodes and topological entropy introduced in^47^. The topological dimension $dimQ_i$ of a node *i* is defined as the number of simplexes of all orders in which the corresponding vertex participates,

1 $$\begin{aligned} dimQ_i \equiv \sum _{q=0}^{q_{max}}Q_q^i \ , \end{aligned}$$

where $Q_q^i$ is determined directly from the ${\Lambda }$ matrix by tracking the orders of all simplexes in which the node *i* has a nonzero entry. Then, with this information, the entropy of a topological level *q* defined as

2 $$\begin{aligned} S_Q(q) = -\frac{\sum _i p_q^i \log p_q^i}{\log M_q} \; \end{aligned}$$

is computed. Here, $p_q^i= \frac{Q_q^i}{\sum _i Q_q^i}$ is the node’s occupation probability of the *q*-level, and the sum runs over all nodes. The normalisation factor $M_q=\sum _i\left( 1-\delta _{Q_q^i,0}\right)$ is the number of vertices having a nonzero entry at the level *q* in the entire graph. Thus the topological entropy (2) measures the degree of cooperation among vertices resulting in a minimum at a given topology level. Meanwhile, towards the limits $q\rightarrow 0$ and $q\rightarrow q_{max}$, the occurrence of independent cliques results in a higher entropy at that level.

In addition, we compute the vector ${\mathbf {f}}= \left\{ f_0, f_1, \ldots f_{q_{max}}\right\}$, which is defined^47^ such that $f_q$ represents the *number of simplexes and faces at the level* *q*. Given that a free simplex of the size $n>q$ has the corresponding combinatorial number of faces of the order *q*, the component $f_q$ thus contains information about the actual number of shared faces between simplexes *at* the level *q*. In this way, with these algebraic-topology measures, we can identify all simplexes with the nodes (brain regions) that form them, as well as how these simplexes interconnect with each other through sharing specific groups of nodes.

### Network structure and hyperbolicity

The underlying topological graph represents the 1-skeleton of the simplicial complex. Using the graph-theory methods^48^, we determine the degree–degree correlations that are relevant to the observed “rich club behaviour” of the hubs in the global connectome^1,27,30,31^. Precisely, for each node in the considered network, the average number of edges of its nearest neighbour nodes is plotted against the node’s degree. The following scaling form is expected

3 $$\begin{aligned} \langle k\rangle _{i:nn} \sim k_i^\mu \ . \end{aligned}$$

Here, the positive values of the exponent $\mu > 0$ indicate the *assortative* correlations, while $\mu < 0$ corresponds to a *disassortative* mixing, and $\mu =0$ suggests the absence of nodes correlations. We analyse the Fc- and Mc-graphs by considering the edges that remain after applying different weight thresholds. The weight distribution *P*(*w*) is determined for the entire core-networks, see Results.

Furthermore, the occurrence of hyperbolicity or negative curvature in the brain graph is a measure of the proximity of nodes (in the graph’s metric space) that facilitates the transmission of signals among different brain regions. We use the 4-point Gromov criterion for the hyperbolic graphs^49^ to determine the hyperbolicity parameter $\delta _{max}$ of these graphs. Precisely, for each 4-tuple of nodes (*A*, *B*, *C*, *D*) in a $\delta$-hyperbolic graph *G*, the ordered relation between the sums of shortest-path distances ${{\mathscr {S}}}\equiv d(A,B)+d(C,D) \le {{\mathscr {M}}}\equiv d(A,C) + d(B,D) \le {{\mathscr {L}}}\equiv d(A,D)+ d(B,C)$ implies that

4 $$\begin{aligned} \delta (A,B,C,D) \equiv \frac{{{\mathscr {L}}} - {{\mathscr {M}}}}{2} \le \delta (G)\; . \end{aligned}$$

It follows from the triangle inequality that the upper bound of $({{\mathscr {L}}}-{{\mathscr {M}}})/2$ is given by the minimal distance $d_{min}\equiv min\{d(A,B),d(C,D)\}$ in the smallest sum ${{\mathscr {S}}}$. Thus, by sampling a large number ($10^9$) 4-tuples of nodes in each graph, and plotting $\delta (A,B,C,D)$ against the corresponding minimal distance $d_{min}$, we obtain $\delta (G)$ as the upper bound of $\delta _{max}=max_G\{\delta (A,B,C,D)\}$.

## Results

### Whole-brain connectomes: identification of hubs from topological dimension

**Figure 1:**
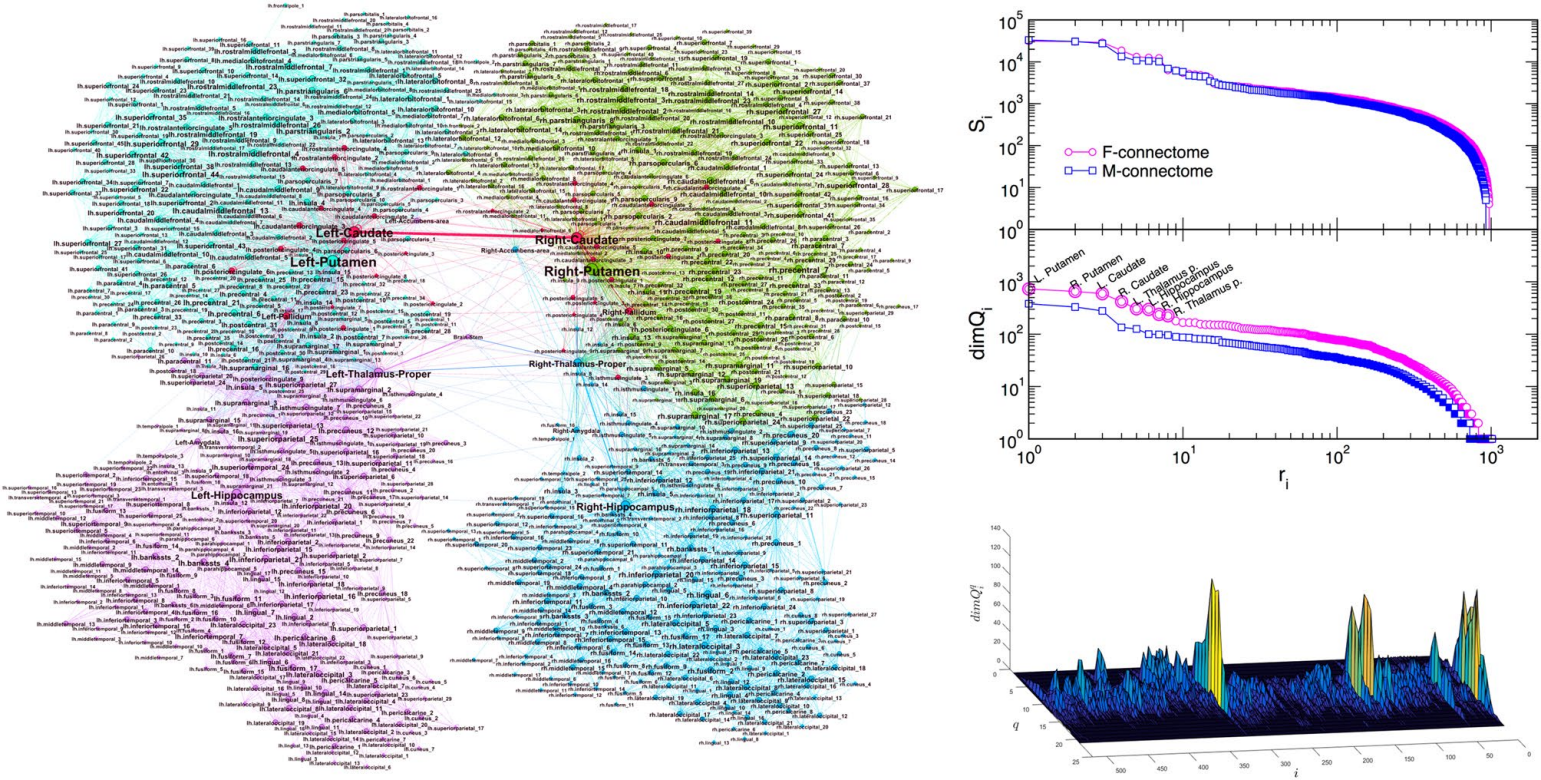
(left) F-connectome, 1000K fibres, with labels as brain areas. (right, top) Ranking of the vertices according to the strength $S_i$, top, and topological dimension $dimQ_i$, lower panel, where eight leading vertices are marked (they also visible as hubs in the network on the left). (right,bottom) The 3D plot of the topological dimension against the topology level *q* and the node’s index *i* for nodes in the F-core graph.

We consider two whole-brain networks, precisely, the F-connectome, which is common for 100 female subjects, and M-connectome, consisting of the edges that are common to 100 male subjects; see Methods and^1^ for more details. For illustration, the F-connectome is shown in the left panel of Fig. 1. Each connectome consists of 1015 nodes as anatomical brain regions (Fig. SI-1). These nodes are interconnected by a particular pattern of edges and organised in mesoscopic communities. For this work, we determine the *global hubs* in the F- and M-connectomes. These are eight top-ranking nodes according to the number of simplexes attached to a node. Based on our work in^1^, we use the corresponding $\Lambda$-matrix for the F- and M-connectomes and identify simplexes of all orders in which a particular node $i=1,2,\ldots 1015$ participates. The node’s topological dimension $dimQ_i$, defined by (1) is then computed. For both connectomes, the node’s ranking distribution by the decreasing topological dimension is shown in the middle right panel of Fig. 1. As the figure demonstrates, the eight top-ranking nodes (marked along the curve for the F-connectome) make a separate group compared to the rest of the curve. These nodes also appear among the first eight ranked topological hubs in the M-connectome, see Table 1.

**Table 1:** Names of eight leading hubs and their ranking order in the female (rank:F) and male (rank:M) connectomes.

rank:F	Hub name	rank:M
1	Left Putamen	1
2	Right Putamen	3
3	Left Caudate	2
4	Right Caudate	4
5	Left Thalamus-Propper	5
6	Left Hippocampus	7
7	Right Hippocampus	8
8	Right Thalamus-Propper	6

For comparisons with other approaches, we also show that these nodes (with altered order) also appear as eight hubs ranked according to the node’s strength $S_i$, defined as the sum of weights of all edges of the node *i*. In this case, the ranking curves of the F- and M-connectomes virtually overlap, see the top right panel in Fig. 1. The lower right panel shows the 3-dimensional plot of the node’s topological dimension over different topology levels *q*. In this plot, the high peaks corresponding to our hubs indicate what orders of simplexes mostly contribute to distinguishing the hubs from the rest of the surrounding nodes. Note that these eight nodes also appear as the leading hubs in several other sorting methods, for example, according to the node’s degree and centrality measures^27,31^. For comparisons with other methods, we also show the names of nodes that rank from 9 to 20 according to the topological dimension in the case of the F-connectome: 
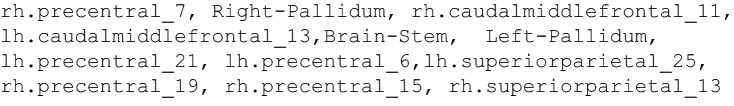
 The nodes listed in the first two rows, except from theBrain Stem, also appear in this ranking range in the M-connectome.

**Figure 2:**
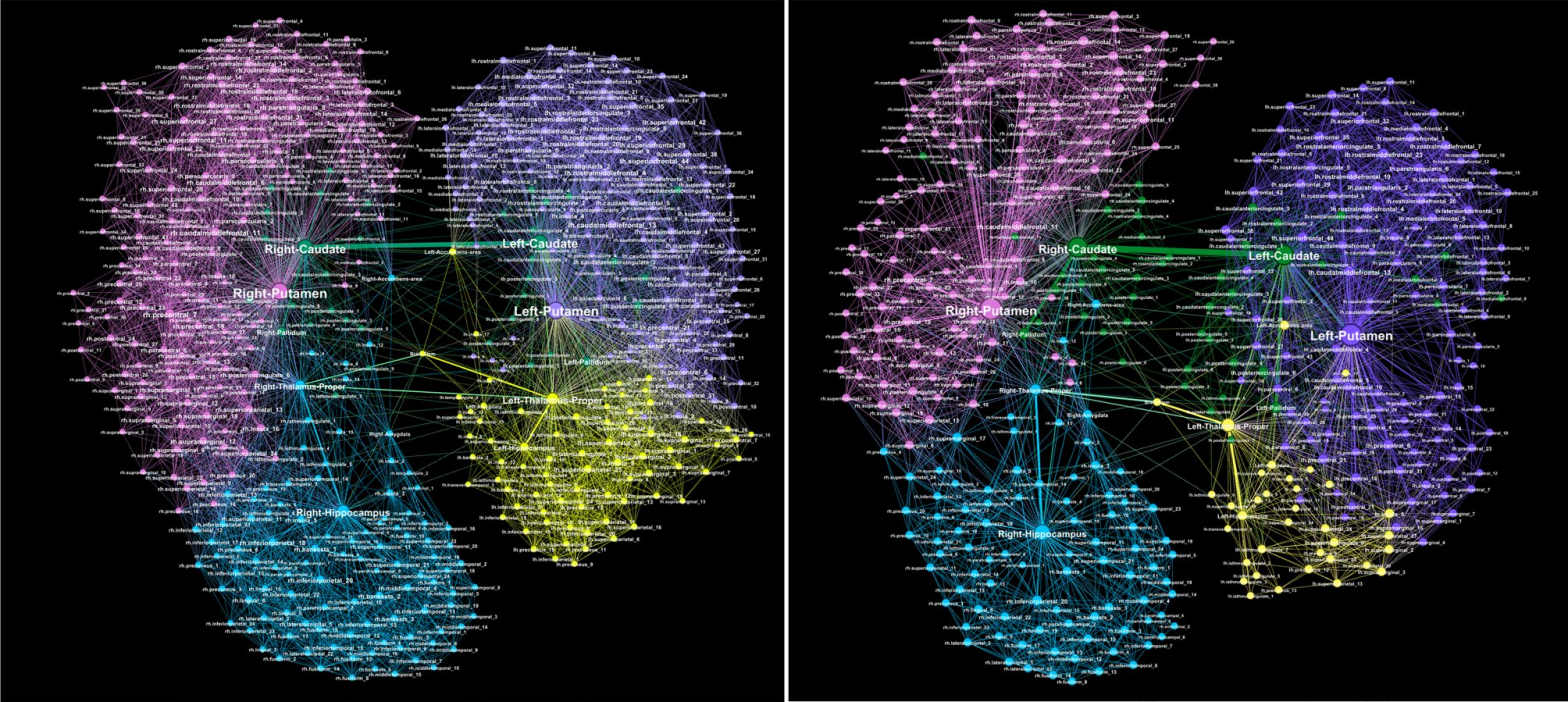
Core networks attached to the eight hubs in the female Fc- (left) and male Mc-connectome (right) from the original full-connectomes data at $N_F=10^6$ fibres tracked and the weight threshold $w_0=4$. The relative size of nodes is proportional to the number of their connections in the core-networks; the node’s labels show the corresponding anatomical brain region, and colours indicate five topological communities.

Next, we consider a reduced network consisting of these hubs and the nodes directly attached to any one of the hubs, as well as the original edges between them in the F- and M-connectomes. The resulting *core networks* termed Fc- and Mc-networks, respectively, are shown in Fig. 2. Note that, by definition, the topological dimension of the hubs is invariant to this network reduction.

### Core networks associated with global hubs in the female and male connectomes

**Figure 3:**
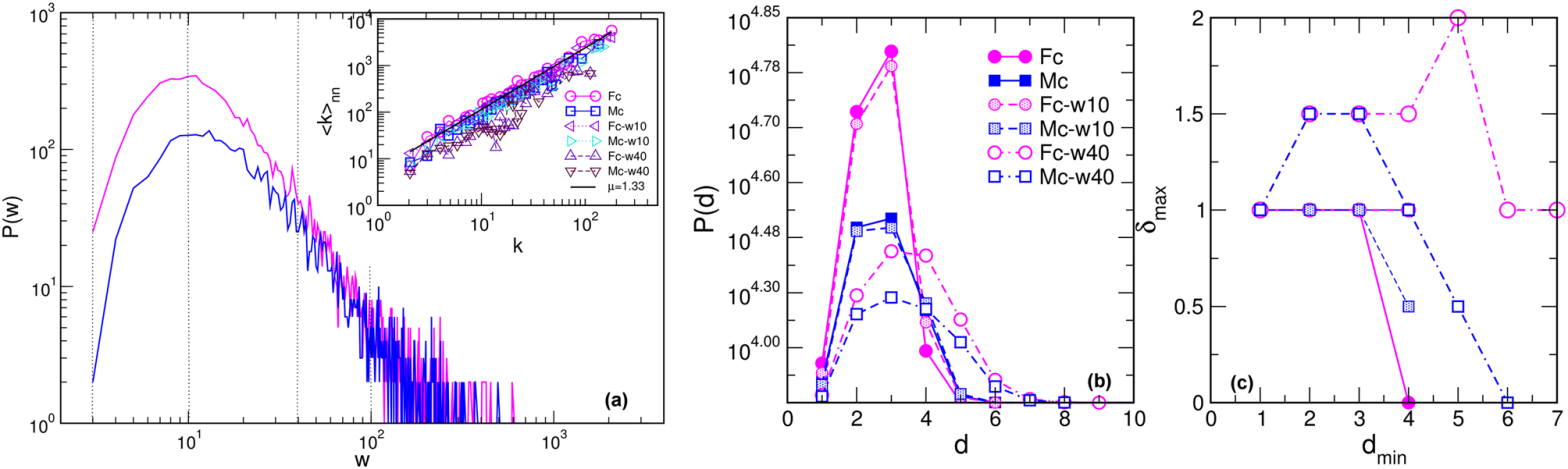
(**a**) Histogram of the weights of edges in the core Fc -and Mc-networks of the corresponding female and male connectomes, main panel; Inset: the assortativity plots of nodes in the core Fc- and Mc-networks for the weight threshold $w_0=4,$ 10, 40, and 100, respectively, indicated by dotted vertical lines in the main panel. (**b**) Distribution of distances *P*(*d*) against the shortest path distance *d* and (**c**) the hyperbolicity parameter $\delta _{max}$ against the shortest distance $d_{min}$ for the core Fc- and Mc-networks shown in Fig. 2, and these networks for two larger threshold weights, indicated in the legend.

The extracted core Fc- and Mc-networks represent the part of the corresponding connectome in which the global hubs perform their function. Here, we explore in detail the structure of the core networks in the female and male connectomes. Furthermore, we analyse how the structure depends on the weights of the edges. The histogram of the weights is shown in Fig. 3a for both Fc- and Mc-networks. As Fig. 2 demonstrates, these core networks exhibit a similar community structure. Precisely, each community in the Fc- and similarly in Mc-connectome is a part of the global connectome community, cf. Fig. 1. This fact suggests that, in both connectomes, the core network reaches to all parts of the brain. Meanwhile, it contains a smaller number of nodes (517 nodes in the Fc- and 418 in the Mc-network, respectively), and a considerably smaller number of connections compared to the whole connectome. Thus, the node’s assortativity changes as compared to the whole network. As the inset to Fig. 3 shows, the hubs mix in line with other vertices in the core graphs, while they make a separate group when the whole connectomes are considered^1^. This assortative dependence emphasises the robustness of core networks with respect to the hierarchical transmission of information among brain regions^50^.

### Topology of core networks depending on the weights of edges

**Figure 4:**
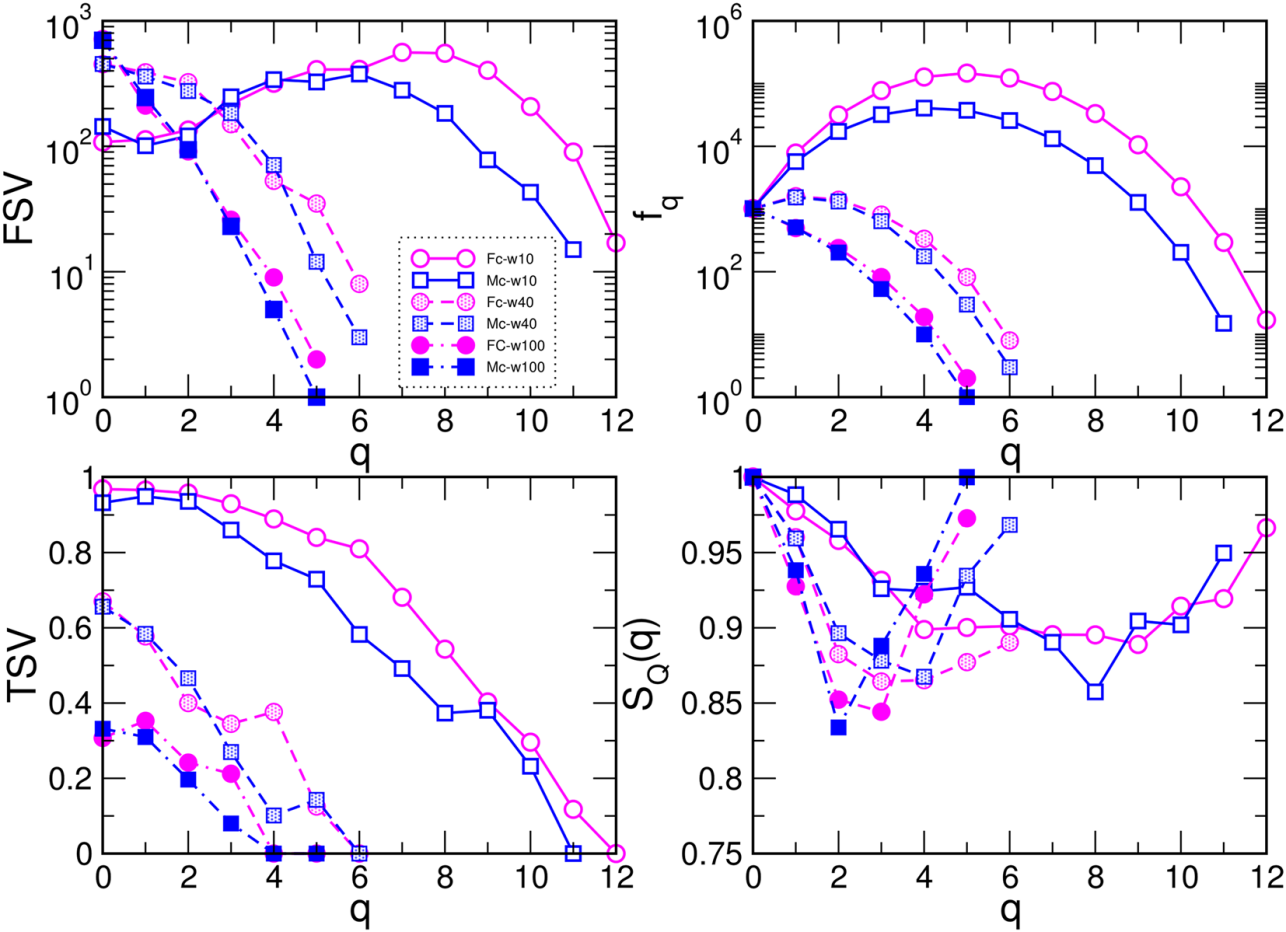
The first (FSV) and third (TSV) structure vectors, the number of simplexes and faces $f_q$, and the topological entropy $S_Q(q)$ against the topology level *q* in the core Fc- and Mc-networks with the edges of weights above the threshold $w_0=10$, 40, 100.

**Figure 5:**
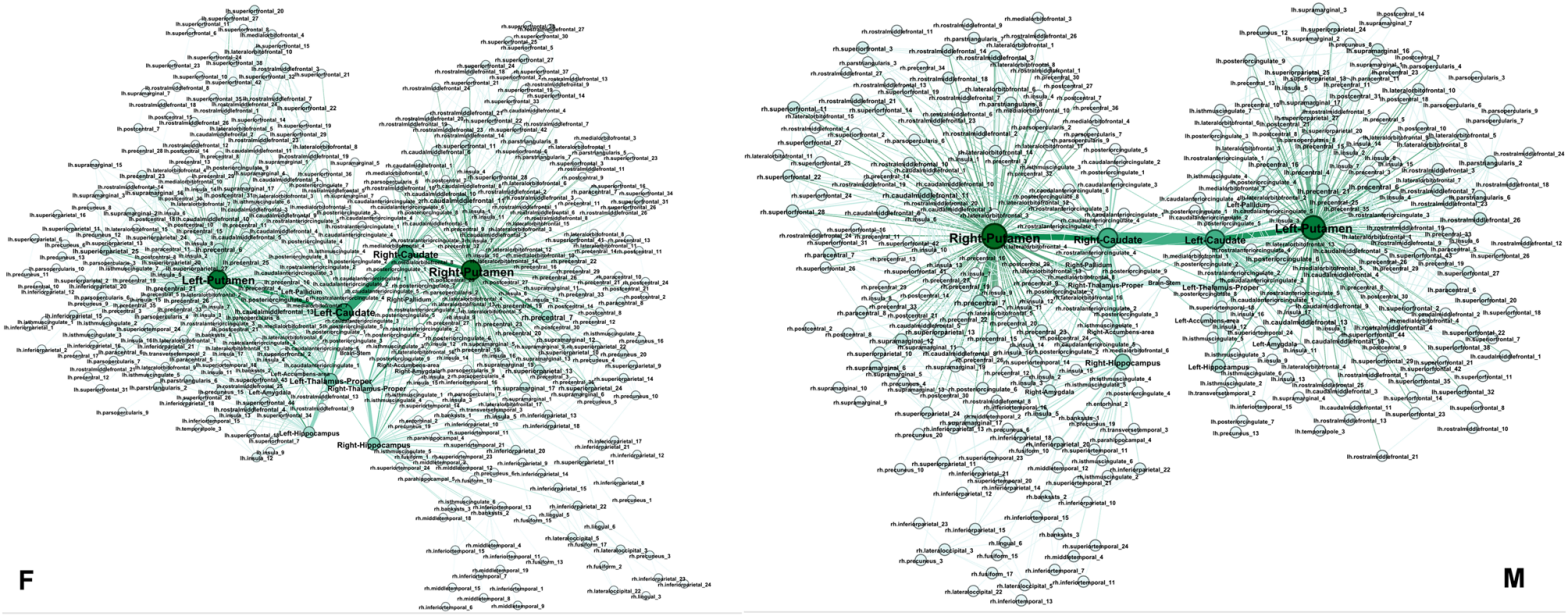
Core networks with the weights of edges above $w_0=40$ for the female (left) and male (right) connectomes. Labels of nodes indicate the affected brain regions.

Using the approaches described in Methods, we determine several algebraic-topology measures to characterise the structure of simplicial complexes as well as the underlying topological graphs in the core Fc- and Mc-networks. These results are summarised in Figs. 3 and 4. Apart from a different number of nodes and edges that comprise the Fc- and Mc-networks, we note that both of them are heterogeneous concerning the weight of edges, resulting in the broad log-normal distributions in Fig. 3a. Therefore, we obtain different structures when the edges over a given threshold weight, $w_0$, are considered. By gradually increasing the threshold $w_0=10$, 40, 100, we show how the network properties change. More precisely, by removing the edges below the threshold, the network’s diameter increases, and the distribution of the shortest-path distances change the shape. Eventually, a larger cycle can appear, resulting in the increased value of the hyperbolicity parameter, as shown in Fig. 3b,c. Meanwhile, the reduced networks preserve the assortative mixing among the nodes, see the inset to Fig. 3a. At the same time, the order of simplicial complexes gradually reduces from $q_{max}=12$, in the case of $w_0=10$, to $q_{max}=5$ when edges over the threshold $w_0=100$ are retained. The number of simplexes of the order $q=0,1,2\ldots q_{max}$, given by the FSV, and the ways that they interconnect, the TSV, change the functional dependence of *q*, as shown in Fig. 4, while at the same time reducing the difference between the Fc- and Mc-structures, cf. Fig. 5. The number of simplexes and faces at the *q*-level, $f_q$, and the topological entropy, $S_Q(q)$, follow a similar tendency. Moreover, the topological entropy measure shows a pronounced minimum, indicating the geometrical forms through which the nodes mostly interconnect. For example, in the case of $w_0=100$, the minimum appears at $q=2$ (triangles) in the Mc-, and $q=3$ (tetrahedrons) in the Fc-networks, respectively. Figure 5 illustrates the remaining structures of the Fc- and Mc-networks when the weight threshold $w_0=40$ is applied.

**Figure 6:**
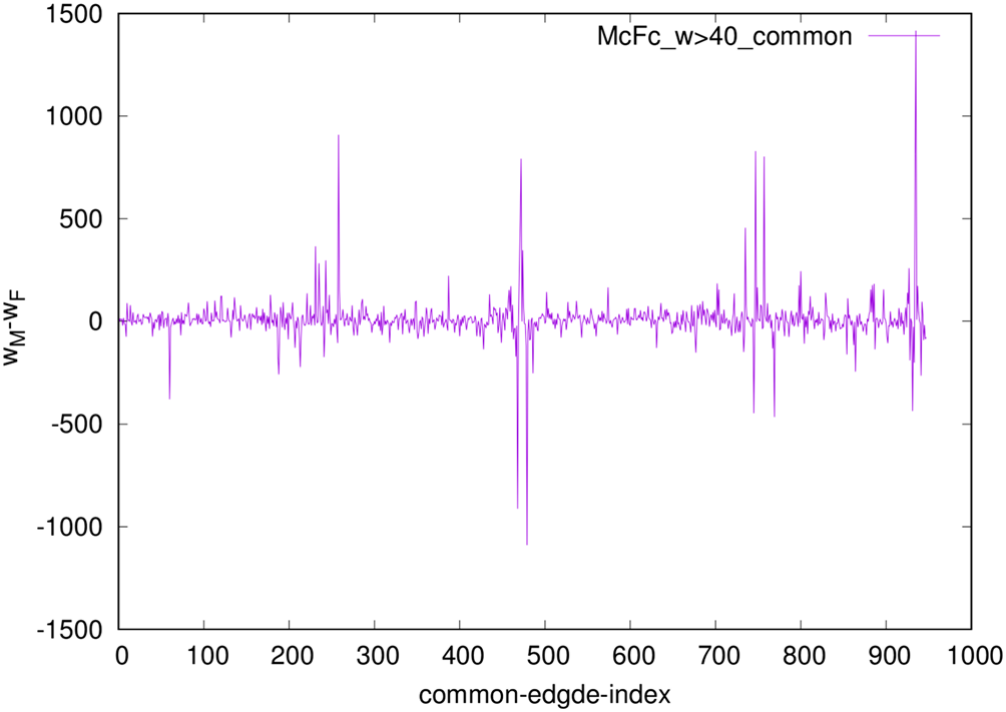
The weight difference $w_M-w_F$ of the common edges, indexed from 1 to 948, in the Fc- and Mc-core networks in Fig. 5 with the edges weight over 40.

An edge-to-edge comparison between the core Fc- and Mc-networks with the threshold weight $w_0=40$, shown in Fig. 5, revealed 948 edges that appear in both of them. Besides, the core Mc-network has 204 unique edges that are not present in the Fc-network with this threshold value, while the Fc-network has 419 such edges that are not seen in the corresponding Mc-network. Moreover, the weight difference among the common edges varies, as shown in Fig. 6. For example, the pairs of nodes that make up 16 edges with a large difference $|w_M-wF| >300$ are listed in Table 2.

**Table 2:** List of edges with a massive weight difference $|w_M-wF| >300$ in the female and male core networks.

ID	Source	ID	Destination	Weight $w_M$	Weight $w_F$
51	rh.$\hbox {parsopercularis}_-$2	504	Right-Putamen	601	980
151	rh.$\hbox {precentral}_-$9	504	Right-Putamen	740	375
159	rh.$\hbox {precentral}_-$7	504	Right-Putamen	2453	1544
502	Right-Thalamus-Pr.	1008	Left-Thalamus-Pr.	809	1721
503	Right-Caudate	504	Right-Putamen	3340	2843
503	Right-Caudate	505	Right-Pallidum	3072	2280
503	Right-Caudate	507	Right-Hippocampus	937	591
503	Right-Caudate	1009	Left-Caudate	7122	8211
651	lh.$\hbox {precentral}_-$21	1010	Left-Putamen	1220	765
654	lh.$\hbox {precentral}_-$16	1008	Left-Thalamus-Pr.	137	584
654	lh.$\hbox {precentral}_-$16	1010	Left-Putamen	1460	631
657	lh.$\hbox {precentral}_-$4	1010	Left-Putamen	1796	993
661	lh.$\hbox {precentral}_-$6	1010	Left-Putamen	618	1084
1008	Left-Thalamus-Pr.	1013	Left-Hippocampus	2481	2917
1009	Left-Caudate	1010	Left-Putamen	3174	2362
1009	Left-Caudate	1011	Left-Pallidum	3222	1805

## Discussion and conclusions

In line with the latest trends in complexity science, we have studied weighted higher-order structures in human connectomes based on the empirical data from the Human Connectome Project. Specifically, by extending the work in^1^, we have analysed the structure of simplicial complexes in the core networks surrounding eight topological hubs. This analysis enabled us to identify brain regions participating in simplexes of different orders that are attached to hubs as well as their hierarchical organisation, which manifests in sharing common subgraphs. In this context, we have also provided new evidence for (dis)similarity between female and male core graphs.

The hubs are accordingly determined as the top-ranking nodes with the highest topological dimension (the number of simplexes attached). Remarkably, they coincide with the hubs determined by several other graph-theoretic measures, representing the central brain regions known to enable complex communication between different parts of the brain^11^. By parallel analysis of the female and male consensus connectomes, we have extracted the corresponding core segments, here termed the Fc- and Mc-networks, in which the brain hubs perform their function. Both in the Fc- and Mc-networks, except for the differences in the size and structure of simplicial complexes, simplexes attached to eight leading hubs reach to almost all parts of the brain. Besides, these core networks have a similar small hyperbolicity parameter in analogy to the complete connectomes studied in^1^. At the graph level, corresponding to 1-skeleton of the simplicial complexes, the nodes in these core networks exhibit assortative mixing, consistent with the “rich-club” structure of hubs previously studied in^27^.

Further, we have demonstrated that these core networks are heterogeneous concerning the weights of edges and they possess different weight-dependent organisations. Consequently, their structure simplifies with the increased weight threshold, eventually reducing at significant thresholds to the 6-clique structure. At the same time, by disregarding the edges below the imposed weight threshold, we determine changes in the structure of the underlying topological graph. It is readily manifested in the occurrence of larger distances among nodes and with them related subjacent graphs, e.g., longer cycles, that are compatible with an increased hyperbolicity parameter. Interestingly, these six nodes 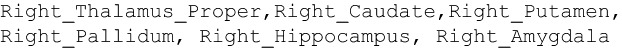 make up a remaining 6-clique structure in both female and male core networks. We have found another 6-clique in the female core network, i.e., 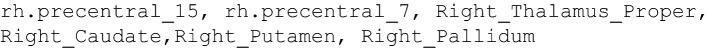 including two additional nodes that belong to the precentral gyrus, part of the primary motor cortex. As mentioned above, these two nodes appear among the first twenty ranked nodes. The study^27^ ranked high the “Right.precentral” node in this region according to the strength among a total of 82 brain region. In both core networks, the identity of the affected brain regions, as well as the variation of the weights along the commonly present edges, illustrates further differences between the female and male connectomes at the level of hubs.

Our results revealed that the core networks surrounding the eight leading hubs in human connectomes extend to different parts of the brain by connecting them through weighted simplexes of different orders. In the context of higher-order interactions, these findings can contribute to better understanding the pattern of connections that enable the brain hubs to perform their role in the female and male connectomes. Besides, the revealed detailed structure of simplicial complexes and the identified brain regions that take part in them can facilitate the desired simplex-based dynamics modelling of the brain functions.

## Supplementary information

Supplementary Information 1

## Data Availability

All data used in this work are available from the Budapest reference connectome 3.0, https://pitgroup.org/connectome/.
