# Supplementary material for: The topology of higher-order complexes associated with brain hubs in human connectomes
Source: Sci Rep. 2020 Oct 14;10:17320. doi: 10.1038/s41598-020-74392-3 (PMC7560876; doi:10.1038/s41598-020-74392-3)
Supplement: Supplementary file 1 — Supplementary Information 1 [file 41598_2020_74392_MOESM1_ESM.pdf]

Supplementary Information for
The topology of higher-order complexes associated with brain hubs in human connectomes

Miroslav Andjelković[a,c], Bosiljka Tadić[a,b], Roderick Melnik[d,e]
[a]*Department of Theoretical Physics, Jožef Stefan Institute, Jamova 39, Ljubljana, Slovenia*
[b]*Complexity Science Hub, Josefstaedter Strasse 39, Vienna, Austria*
[c] *Department of Thermal Engineering and Energy, Vinča Institute of Nuclear Sciences - National Institute of the Republic of Serbia, University of Belgrade, 11000 Belgrade, Serbia*
[d]*MS2Discovery Interdisciplinary Research Institute, M2NeT Laboratory and Department of Mathematics Wilfrid Laurier University, Waterloo, ON, Canada;* [e]*BCAM - Basque Center for Applied Mathematics, Alameda de Mazarredo 14, E-48009 Bilbao, Spain*

Contents

Figure SI-1: High-resolution image of the female consensus connectome at 1000000 tracked fibres with 1015 labelled brain regions, weighted edges, and colour-coded communities:

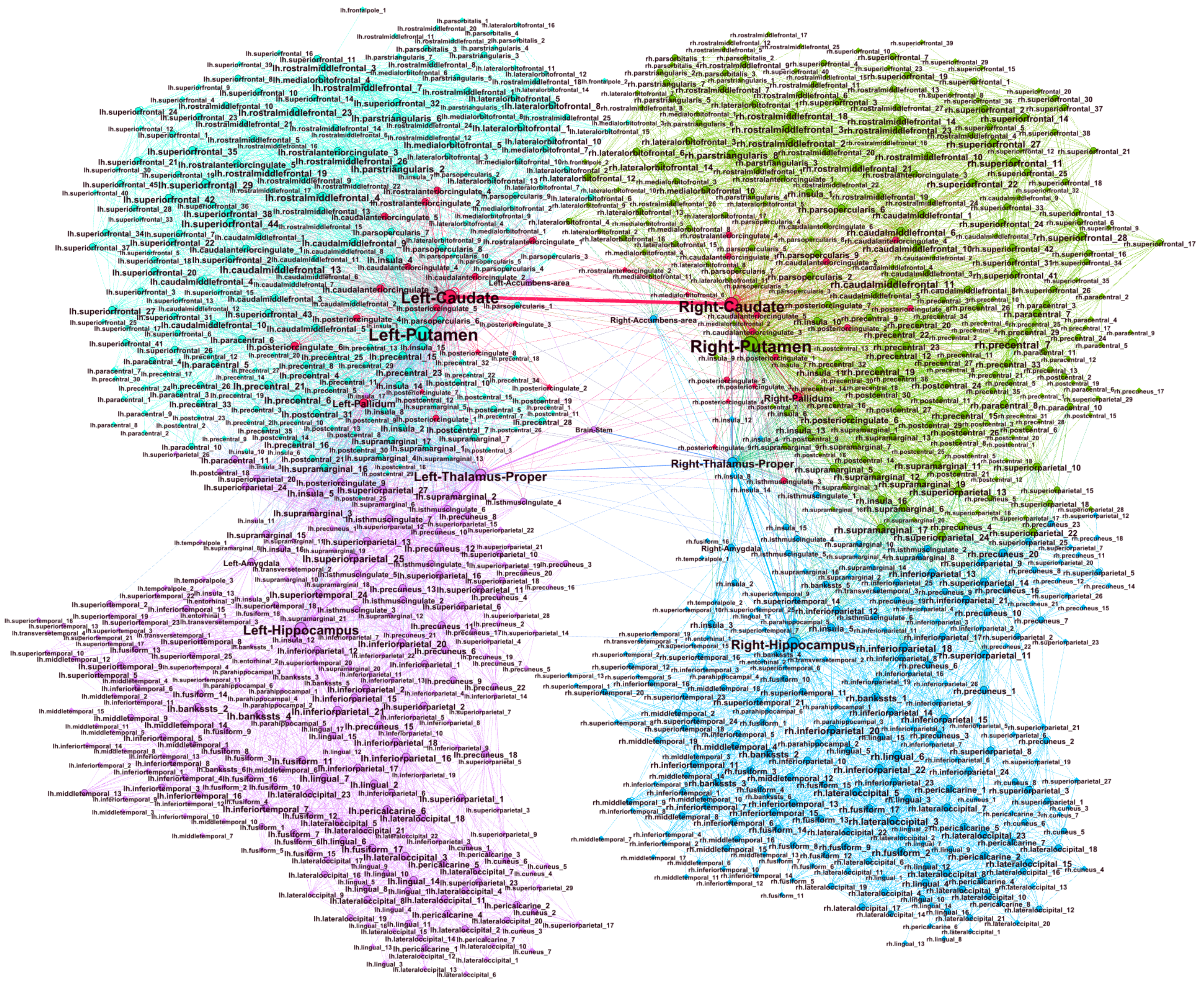
Left-Caudate
Right-Caudate
Left-Putamen
Right-Putamen
Left-Accumbens-area
Right-Accumbens-area
Left-Pallidum
Right-Pallidum
Brain-Stem
Left-Thalamus-Proper
Right-Thalamus-Proper
Left-Amygdala
Right-Amygdala
Left-Hippocampus
Right-Hippocampus

FIG. SI-1:
